# Neurocognitive disorders in the elderly: altered functional resting-state hyperconnectivities in postoperative delirium patients

**DOI:** 10.1038/s41398-021-01304-y

**Published:** 2021-04-12

**Authors:** Jeanne M. Winterer, Kwaku Ofosu, Friedrich Borchers, Daniel Hadzidiakos, Florian Lammers-Lietz, Claudia Spies, Georg Winterer, Norman Zacharias

**Affiliations:** 1 Department of Psychiatry and Psychotherapy (CCM), Charité-Universitätsmedizin Berlin, Corporate Member of Freie Universität Berlin, Humboldt-Universität zu Berlin and Berlin Institute of Health, Berlin, Germany; 2 Department of Anesthesiology, Charité (CVK, CCM)–Charité-Universitätsmedizin Berlin, Corporate Member of Freie Universität Berlin, Humboldt-Universität zu Berlin and Berlin Institute of Health, Berlin, Germany; 3 Pharmaimage Biomarker Solutions GmbH, Berlin, Germany

**Keywords:** Predictive markers, Neuroscience

## Abstract

Postoperative delirium (POD) represents a confusional state during days/weeks after surgery and is particularly frequent in elderly patients. Hardly any fMRI studies were conducted to understand the underlying pathophysiology of POD patients. This prospective observational cohort study aims to examine changes of specific resting-state functional connectivity networks across different time points (pre- and 3–5 months postoperatively) in delirious patients compared to no-POD patients. Two-hundred eighty-three elderly surgical patients underwent preoperative resting-state fMRI (46 POD). One-hundred seventy-eight patients completed postoperative scans (19 POD). For functional connectivity analyses, three functional connectivity networks with seeds located in the orbitofrontal cortex (OFC), nucleus accumbens (NAcc), and hippocampus were investigated. The relationship of POD and connectivity changes between both time points (course connectivity) were examined (ANOVA). Preoperatively, delirious patients displayed hyperconnectivities across the examined functional connectivity networks. In POD patients, connectivities within NAcc and OFC networks demonstrated a decrease in course connectivity [max. *F* = 9.03, *p* = 0.003; *F* = 4.47, *p* = 0.036, resp.]. The preoperative hyperconnectivity in the three networks in the patients at risk for developing POD could possibly indicate existing compensation mechanisms for subtle brain dysfunction. The observed pathophysiology of network function in POD patients at least partially involves dopaminergic pathways.

## Introduction

Prevalence of delirium is extremely high among hospitalized individuals older than 85 years, whereas it depends on the individual´s characteristics, setting of care, and screening sensitivity^1–6^. It develops over a short period of time (hours to days) and can fluctuate over the course of a day. Delirium is usually caused by one or several serious medical conditions, likely triggered by an acute illness, hospitalization, or surgery^7^. Delirium in the elderly is repeatedly underestimated and frequently misdiagnosed^4,8,9^. Contrary to Alzheimer’s disease, a delirium itself can be reversed up to a certain degree^10,11^, with cognitive deficits possibly persisting up to one year^12^.

Postoperative delirium (POD) affects about 10–70% of surgical patients older than 65 years^13^, representing a frequent complication among elderly surgical patients^14–16^. Delirium incidence also is frequently seen in patients with specifically hip fractures and cardiac surgery^17,18^. It can emerge up to one week postoperatively or until discharge and last for several days to weeks^19–21^. It has been reported that delirium duration predicts long-term postoperative neurocognitive disorder and worsening of dementia^22,23^. Although mortality and morbidity rates among POD patients are still debatable, patients do face prolonged hospital stays resulting in higher hospital costs^24–28^. It is expected that POD will be of increasing concern for the society, as the number of surgical interventions will continue to rise since our society is constantly growing older.

As functional connectivity between brain areas was shown to be associated with complex cognitive functioning^29,30^ functional magnetic resonance imaging (fMRI) presents a potential noninvasive tool to unravel the pathophysiology of postoperative delirium. Until now research in this field rarely applied fMRI and the number of studied patients was generally small^31^. Up to now, one of the first fMRI studies on delirium was conducted in South Korea and published in 2012 by Choi et al.^32^. Using resting-state fMRI (rs-fMRI) and examining the default mode network (DMN), they found that functional connectivity of dorsolateral prefrontal cortex (DLPFC) and posterior cingulate cortex (PCC) as well as for PCC and precuneus differ between patients and control participants during an episode of delirium. Additionally they found transient changes in functional connectivities between intralaminar thalamic and caudate nuclei with other subcortical regions during an episode of delirium. The same group^33^ also demonstrated with rs-fMRI disruptions of global network integration and efficiency among delirious patients and the network integration decreasing the longer the delirium. In two more recent rs-fMRI studies, the group from South Korea^34,35^, conducted preoperative fMRI in *n* = 25 patients who subsequently developed delirium (POD) and *n* = 32/33 patients without delirium. *N* = 14 POD patients also had follow-up fMRI scans. Among others, they first illustrated increased amplitude of low-frequency fluctuation (ALFF) in the DLPC preoperatively. Second, during delirium, they conducted voxel-based connectivity analyses, in which two seeds in the DMN (PCC, medial prefrontal cortex (mPFC)) and connections between 11 subcortical regions were also examined. Several replicative findings were obtained compared to their earlier study. Increased connectivity between the PCC and DLPFC during delirium was equally evident in both studies, which led them to suggest that delirium is characterized by diminished anti-correlation between the DMN and task-positive regions. Also, reduced connectivity between subcortical regions was another replicative finding, which they considered to be compatible with the notion that stable connectivity related to cholinergic/dopaminergic neurotransmission and the proper function of the ascending reticular activating system.

Our study intends to gain further insight into the longitudinal course of delirium development conducting rs-fMRI scans before and three to five months after an unspecific elective surgery. In particular, we were interested to answer the question if POD patients differ from no-POD patients preoperatively and if lasting course changes are seen in POD patients compared to no-POD patients. Different from the approach of the group from South Korea, our focus was on studying functional connectivities associated with the hippocampus, the nucleus accumbens (NAcc), and the orbitofrontal cortex (OFC) as seed regions. We selected these functional connectivities for the following reasons. Structural abnormalities in the striatum and hippocampus were associated with delirium while being related to the severity of perceptual disturbances, as well as attentional and cognitive deficits^34,36,37^. In structural imaging analyses of patients with Alzheimer’s disease, it was demonstrated that the striatal nucleus accumbens (NAcc) and hippocampus to be related to cognitive impairments, these patients also present resting-state functional disconnectivity with the hippocampus, subcortical and prefrontal areas^38–44^. In the OFC, part of the prefrontal cortex, structural abnormalities are associated with symptoms of psychosis similarly to features of delirium^45–47^. Last but not least, these functional connectivities are related to the dopaminergic system crucial to delirium development and cognitive decline among elderly^10,48,49^.

Based on these functional connectivities, our study aims to identify pre-/post-operatively connectivity changes specifically related to the course of delirium in an explorative manner. The rs-fMRI scans, performed immediately pre- and three to five months postoperatively, are used to verify the following hypotheses: Considering the three functional connectivities, first, since POD has been shown to be related to reduced preoperative cognitive function^50^, compared to the no-POD group, POD patients will demonstrate differences in preoperative functional connectivity. Second, the disruption in global network integrity induced by the surgery will lead to a decrease in connectivity from pre-to post-operative among POD patients.

## Materials and methods

This prospective observational cohort study was conducted as part of the EU-funded BioCog-study (Biomarker Development for Postoperative Cognitive Impairment in the Elderly, www.biocog.eu) and will be presented as a primary report of the data of the neuroimaging sub-cohort measured in Berlin. The major goal of the BioCog study was to identify and develop biomarkers for the prediction of postoperative cognitive dysfunction. The study was approved by the local ethics committees and registered on clinicaltrials.gov (NCT02265263). Detailed information about the assessment procedure and recruitment can be found in the supplement (see Supplemental Figs. 1 and 2).

### Participants

Participants had to be at least 65 years old, undergo elective major surgery (>60 min of time; procedures within body cavity, orthopedic operations, cardiac surgery, or head/neck operations), a MMSE (Mini-Mental State Examination) score of ≥23, and no history of prior neuropsychiatric morbidity. They had to be of Caucasian decent and be able to give informed consent preoperatively. Participants were excluded from the study if there were any signs of deafness, current centrally acting medication (e.g., antidepressants or tranquilizers), homelessness or institutional stay or participating in another study.

The study was conducted at two clinical academic sites (Charité–University Medicine Berlin, Germany; University Hospital of Utrecht, Netherlands)^4^. For the purpose of this fMRI study, only patients from Berlin were selected. With a population of about 6 million inhabitants in the Berlin area and half of all surgical interventions being performed at the Charité University Hospital, the study cohort is thought to adequately represent the elderly population in the region. Specific information for the determination of the sample size can be found in the supplement methods and Supplemental Fig. 2. From the originally included sample of *N* = 747, 431 patients obtained 3 month follow-up assessments. After availability and quality check of MRI data (i.e., preprocessing, maximum motion, invalid scans) and consideration of incomplete delirium scores (*n* = 4), 283 patients were left for the preoperative analysis. Of those, 178 patients underwent both pre- and postoperative MRI scans, presenting the basis for our course connectivity analyses. A matched sample comparison gathered from the same study, with age, sex, and MMSE as matching parameters, was conducted for validation purposes (POD = 46; no-POD = 46).

### Clinical assessments

Demographic parameters (age, sex, educational background (International Standard Classification of Education; ISCED)), body mass index (BMI), American Society of Anesthesiologists physical status classification (ASA), and MMSE were documented preoperatively. Pre- and post-operative assessment included a computerized neurocognitive test battery (Cambridge Neuropsychological Test Automated Battery (CANTAB)), paper-pencil tests (TMT-A & B, Grooved Pegboard), multiple questionnaires, blood sampling, and MRI/EEG scanning. Trained medical staff collected intra- and post-operative data (i.e., surgical time, intensive care unit (ICU) and hospital duration) and they also assessed postoperative delirium based on CAM (-ICU), Nu-DESC, and DSM-5. Delirium questionnaires were completed twice a day one day preoperatively, and during their hospital stay after the surgery (average of 7 days). With consent of the neuropsychologists, trained nurses and doctoral students performed the assessments. In case of delirium incidence, at least one of the following questionnaires stated its presence.

#### DSM-V (Diagnostic and Statistical Manual of Mental Disorders 5th edn)

Presence of delirium is met when the following certain criteria apply: the patient expresses a disturbance in attention and awareness, which developed over a short period of time and has a fluctuating nature. Further, an additional cognitive or perceptual deficit is present (i.e., memory, language, visuospatial abilities). It is not better explained by another neurocognitive disorder or comatose state. The disturbance has to be not associated with substance intoxication or withdrawal.

#### CAM (confusion assessment method) and CAM-ICU (intensive care unit)

Both versions are operationalized on the DSM-IIIR criteria for delirium. Whereas the long version of the CAM retrieves delirium with ten items (acute change in mental status, inattention, altered level of consciousness, disorganized thinking, memory impairment, perceptual disturbances, psychomotor agitation, psychomotor retardation, altered sleep-wake-cycle), the short and ICU version are reduced to four features (acute onset and fluctuating course, inattention, disorganized thinking, and altered level of consciousness). Across all three versions, features one and two have to be displayed, with feature 3 or 4 being present. A score of 3 indicates POD. The CAM-ICUs sensitivity is about 81% and specificity 96%^51^.

#### Nu-DESC (Nursing Delirium Screening Scale)

This custom-made scale for nurses’ presents an extension of the Confusion Rating Scale (CRS) based on DSM-IV criteria. Symptom criteria, rated from 0 to 2, are split into disorientation, inappropriate behavior, inappropriate communication, illusion/hallucinations, and psychomotor retardation. The presence of two or more symptoms relates to delirium. Its sensitivity is about 83% and specificity 81%^51^.

### Brain image acquisition and analysis

Participants underwent MRI one day preoperatively and ~3 to 5 months postoperatively (*M* = 113 days, SD = 29 days) with a median hospital length of 6 days. Imaging data were collected at the Berlin Center for Advanced Neuroimaging (BCAN) with a Siemens 3 T Magnetom Trio MR scanner using a 12-channel head coil. Resting-state fMRI data were collected simultaneously with electroencephalography (EEG) using a 32-channel EEG setup (BrainAmp MR, Brain Products GmbH, Gilching, Germany). The findings of the EEG analysis will not be part of the current publication and will be published elsewhere. Parameters of the imaging acquisition were for rs-fMRI: EP2D-BOLD, 8 min duration, 238 slices (first 10 were discarded to reach equilibrium of spin history), descending slice order, voxel size 3 x 3 x 3 mm³, TR/TE = 2000/30ms, eyes closed. For structural analysis a MPRAGE sequence was used with following parameters: 192 sagittal slices, 1 x 1 x 1 mm³ voxel size, TR/TE = 2500/4.7 ms.

### Preprocessing

The acquired MR data were processed using MATLAB-based CONN connectivity toolbox V17.f (Gabrieli Lab, Massachusetts, USA^52^). The preprocessing steps were as following: realignment, centering, slice-timing correction, outlier identification via a scrubbing process (using Artifact Detection Tool, ART^52^), gray/white matter/cerebral spinal fluid (CSF) segmentation and normalization of the functional and structural images to Montreal Neurological Institute (MNI) space, as well as co-registration of the individual functional and anatomical images and smoothing (Gaussian kernel of FWHM = 8 mm). Using an anatomical component-based noise correction method (anatomical CompCor^53^), data were denoised with the six head motion parameters estimated by the realignment process and the scrubbing covariates. This method extracted a representative noise signal from white matter regions and from CSF and removed anything from the BOLD signal that correlates with those noise components and the aforementioned covariates for every voxel. Thus, it was possible to remove confounding sources of signal variation, which survived the preprocessing process. A band pass filter of 0.01 to 0.1 Hz was applied to the data.

### ROI-to-ROI analysis

For the ROI-to-ROI analysis (i.e., region of interest) brain maps of bivariate correlation coefficients were calculated for three different bilateral seed regions known to be relevant in delirium and dementia research (e.g., OFC, MNI ±27, 21, −19^54^; NAcc, MNI ±10, 12, −06^55^; and hippocampus, MNI ±26, −18, −17^56^) defined by the default CONN ROI parcellation using Harvard-Oxford atlas. The connectome rings provide a broad overview of the general amount and nature of significant connections between the single regions (family wise error corrected (FWE), *p* < 0.05).

### Seed-based functional connectivity analysis

For seed-based analysis the same brain maps and atlas of the ROI-to-ROI analyses were used. Herewith, the mean time-course of each bilateral seed regions was correlated with time courses of all other brain voxels. The resulting seed-to-voxel correlations were entered into a second-level general linear model while performing a one sample *t*-test to examine the average effect across all subjects to receive connectivity specific patterns. To account for multiple comparisons uncorrected (*p* < 0.001) voxel-level height threshold and family wise error corrected (FWE; *p* < 0.05), cluster-level extent threshold were used^52^. The resulting significantly positive or negative clusters were served as a map, threshold by *T* = 25 for the OFC and for the hippocampus and *T* = 11 for NAcc, instead of using standard canonical maps for network definition. The resulting maps were used for extracting the individual mean connectivity values of each network. The resulting mean connectivity’s formed the basis for the statistical analyses.

### Statistical analyses

In general, an alpha level of 0.05 for significance testing was applied. Demographic information was compared between the two groups (POD and no-POD participants) with non-parametric Chi-Square test (i.e., sex, ISCED, ASA, benzodiazepine intake immediately before surgery, site of surgery, lacunar infarcts, type of anesthesia) and Mann–Whitney *U*-test (i.e., age, BMI, surgical time, hospital stay length (time from hospital admission to discharge), ICU duration, white matter hyperintensity volume). A *p*-value of <0.05 (two-tailed) was considered statistically significant. In order to validate the results, a matched sample comparison was performed. The whole analysis was conducted with SPSS 23 and Excel. Distributions were also checked for normality and homoscedasticity.

In order to compare the preoperative functional connectivity between the POD and no-POD group while taking covariates into account (sex), partial correlations were conducted with POD incidence as independent variable and connectivity region of interest as dependent variable. Sub-sequential Analysis of Variance (ANOVA) determined the significances. The significant connectivities were the basis for the course connectivity analysis. It would go beyond the scope of this paper to further focus on the course analysis of non-significant preoperative regions.

Difference scores were calculated of the significant connectivity areas of the pre-analysis (postoperative scores minus preoperative scores) displaying the connectivity change (course connectivity). For each area an analysis of variance (ANOVA) compared the course between the two groups with delirium incidence as independent and difference score as dependent variable. The remaining significant connectivity areas served as basis for the analysis of the postoperative connectivity scores.

The length of delirium is defined by the sum of the several NuDesc-measures after the surgery. Hereby, one measure point portrays half a day. The length is calculated by the duration between the incidences of delirium scores (within this period the participants could also score no delirium due to its fluctuating nature). Linear regressions analyzed the degree of associations between the difference scores and the length. An exploratory analysis checks whether the duration is associated with clinical cognitive parameters (see supplement for further information).

The remaining significant connectivity areas (and an additional focus on the hippocampus connectivities) of the course connectivity serve for the analysis of the postoperative scores. Herewith, ANOVAs check for significant differences between the POD and no-POD group.

## Results

### Demographic, clinical, and operative assessments

The preoperative analysis included 283 participants (with 16% POD incidence). Owing to quality checks (quality checks passing both MRI measurements) and loss to follow-ups, the data for the course and postoperative analysis declined to 178 participants. Table 1 provides the demographics and peri-operative parameters with sex, ASA, type of anesthesia, ICU duration, site and time of surgery, and hospital duration (no-POD *Mdn* = 5, POD *Mdn* = 11) being significantly different amongst all mentioned variables between the two groups. Whereas sex was further used as a covariate in the analysis, we do not integrate the well-known predictors of POD ASA and surgical time as they diminish the statistical power and parts of the results (i.e., partial correlations with subsequent analysis of variance). Additionally, the preoperative sample included nine patients demonstrating postoperative hallucinations (no-POD = 3, POD = 6) with three of those remaining in the course/postoperative sample (no-POD = 1, POD = 2). None of the patients took pre- and post- operative neuroleptic medication. Since pain presents a possible influence on the functional connectivity of the nucleus accumbens^57^, we checked whether there were group differences. As this was not the case (statistical values under *p* > 0.05), we assume a similar effect between the groups. With regards to the neurocognitive testing, preoperatively the groups merely differed in their TMT-A performance, with POD patients presenting longer reaction times (see Supplemental Tables S1a/b). 62 patients were in intensive care unit (min = 0.2 days, max = 48.5 days).

**Table 1:** Patient demographics and peri-operative factors.

Parameter of interest	*N* (no-POD/POD)	Total (*n* = 283)	No-POD group (*n* = 237)	POD group (*n* = 46)	*p*-value
Demographics					
Age [years]	237/46	72 (68/75)	69 (68/75)	73 (70/76)	0.105
Male sex	237/46	128 (45.2%)	101 (42.6%)	27 (58.7%)	0.045
BMI [kg/m^2^]	237/46	26.57 (23.99/28.87)	26.57 (24.03/29)	26.72 (23.27/28.45)	0.646
ISCED	205/39				0.123
II		43 (17.6%)	36 (17.6%)	7 (17.9%)	
III		93 (38.1%)	84 (41.0%)	9 (23.1%)	
IV		9 (3.7%)	8 (3.9%)	1 (2.6%)	
V		99 (40.6%)	77 (37.6%)	22 (56.4%)	
ASA score	235/45				0.049
I		6 (2.1%)	4 (1.7%)	2 (4.4%)	
II		195 (69.9%)	168 (71.5%)	27 (60.0%)	
III		78 (27.9%)	63 (26.8%)	15 (33.3%)	
IV		-	-	-	
V		1 (0.4%)	-	1 (2.2%)	
Baseline MMSE		29 (28/30)	29 (28/30)	29 (27/30)	0.278
Benzodiazepine intake immediately before surgery	230/44	39 (14.2%)	31 (13.5%)	8 (18.2%)	0.413
Intra-and post-operative factors					
Site of surgery (intracranial, intrathoracic/ abdominal/pelvic, peripheral)	237/46	4/108/171 (1.4%/38.2%/60.4%)	3/82/152 (1.3%/34.6%/64.1%)	1/26/19 (2.2%/56.5%/41.3%)	0.015
Surgical time [min]	236/46	97 (55/164)	86 (45/140)	198 (95/297)	< 0.001
ICU duration [days]	236/46	0 (0/0)	0 (0/0)	0.4 (0/0.4)	< 0.001
Duration of hospital stay [days]	236/46	6 (3/9)	5 (3/8)	11 (6/22)	< 0.001
Type of anesthesia (general/regional/combined)	236/46	219/14/49 (77.9%/4.9%/17.2%)	188/13/35 (79.7%/5.5%/14.8%)	31/1/14 (67.4%/2.2%/30.4%)	0.030
MRI markers					
Patients with lacunar infarcts	49/6	54 (19%)	48 (20.2%)	6 (13%)	0.730
WMH volume [ml]	231/43	2.08 (0.88/4.61)	1.94 (0.81/4.39)	2.33 (1.36/5.05)	0.264

Median (25/75% percentiles) (non-normal distributed), frequencies in *n* (%).

*POD* postoperative delirium, *BMI* body mass index, *ISCED* International Standard Classification of Education, *ASA* American Society of Anesthesiologists, *MMSE* mini-mental state examination, *ICU* intensive care unit, *WMH* white matter hyperintensity.

### ROI-to-ROI analysis

**Fig. 1:**
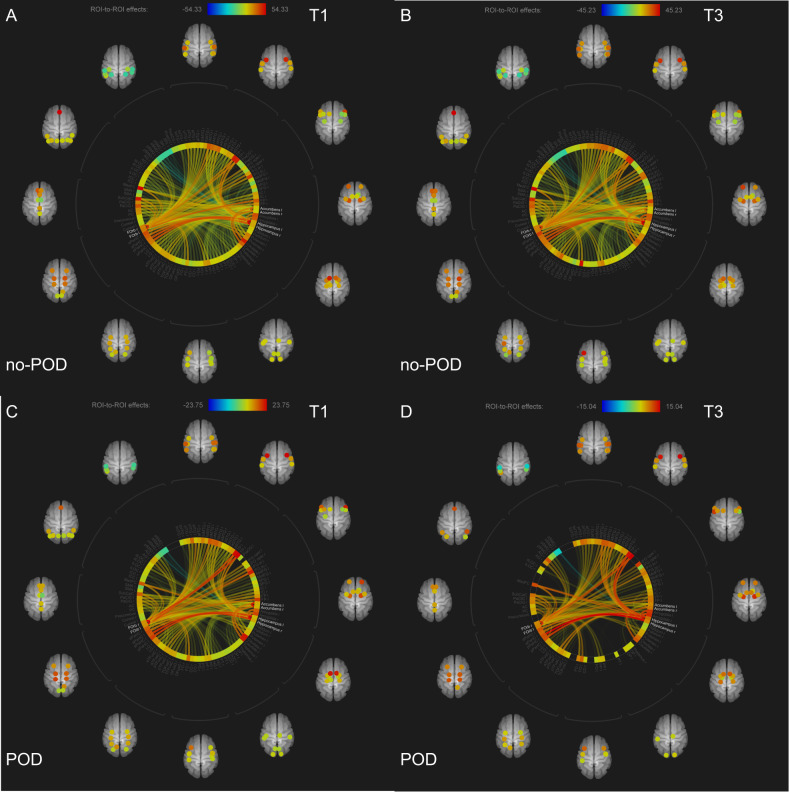
ROI-to-ROI analysis for each time point (T1, T3) and each group including the three seeds (orbitofrontal cortex, nucleus accumbens, hippocampus). **A** Preoperative no-POD connectome. **B** Postoperative no-POD connectome. **C** Preoperative POD connectome. **D** Postoperative POD connectome. Note: Different scales for each connectome due to different effect sizes. POD Postoperative delirium.

**Fig. 2:**
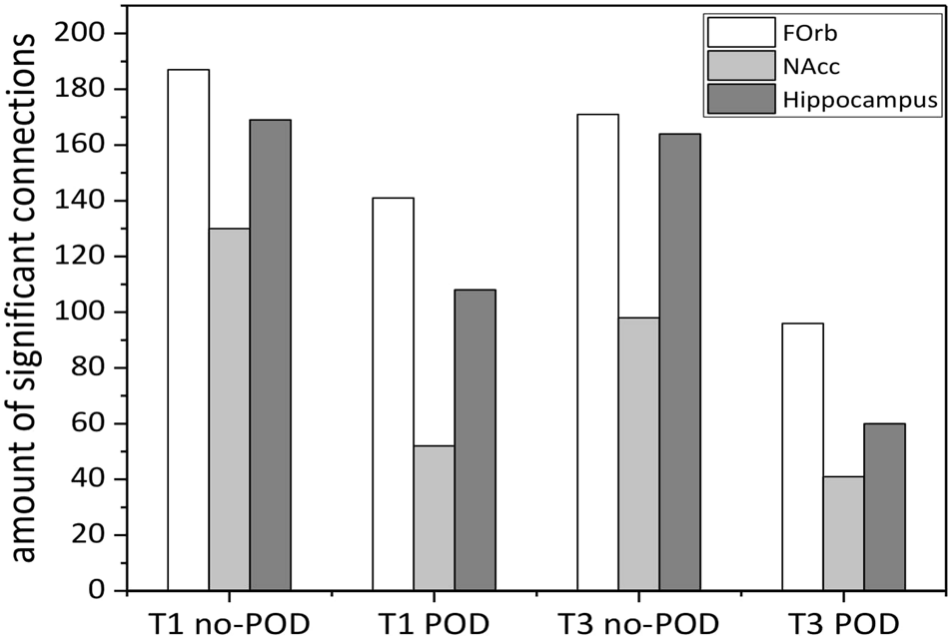
Amount of significant connections (FWE corrected, *p* < 0.05) for each time point and each group including the three seeds (orbitofrontal cortex, nucleus accumbens, hippocampus). FOrb Frontal orbital cortex, NAcc Nucleus Accumbens.

Figure 1 presents the connectome rings for each time point and group. Among no-POD patients, between the pre- and post-operative analysis, there is barely any difference in the amount of connections. Considering the orbitofrontal cortex, it appears to be well connected with the posterior medial temporal gyrus, the temporal pole, inferior frontal triangulate gyrus, subcallosum, paracingulate gyrus, planum polare, and frontal pole. The nucleus accumbens seemed to be well connected with the caudate, supramarginal gyrus, planum polare, paracingulate gyrus, and medial temporal gyrus. The hippocampus appears to be well connected with parahippocampal gyrus, temporal pole, and medial temporal gyrus. Among POD patients, between pre-and post-operative analyses, there is a reduction in connections. Preoperatively, the orbitofrontal cortex occurs to be well connected with the temporal pole, the medial frontal cortex, frontal pole, frontal operculum, the inferior triangulate gyrus, medial temporal gyrus, posterior inferior temporal gyrus, and paracingulate gyrus. The nucleus accumbens was well connected with the caudate, subcallosal, and supramarginal gyrus. The hippocampus was well connected with the parahippocampal gyrus, the amygdala, temporal pole, and medial frontal cortex. Postoperatively, the number of connections was reduced. Comparing both groups (Fig. 2), POD patients demonstrate at both time points less connections and especially the number of hippocampus connections was postoperatively reduced compared to the no-POD group.

### Preoperative connectivity analysis

Partial correlation analyses (with sex as covariate) displayed within every ROI differences in multiple connectivities (Supplemental Tables S2a–c). Considering the OFC, two statistically significant correlations were obtained (right inferior frontal triangulate gyrus (*r* (280) = 0.14, *p* = 0.021) and the right inferior temporal gyrus (*r* (280) = 0.12, *p* = 0.037)). Significant results were identified in five of the NAcc functional connectivities: left accumbens (*r* (280) = 0.20, *p* = 0.001), right accumbens (*r* (280) = 0.17, *p* = 0.004), right Heschl’s gyrus (*r* (280) = 0.16, *p* = 0.007), left planum polare (*r* (280) = 0.13, *p* = 0.026), and right planum polare (*r* (280) = 0.14, *p* = 0.018). Furthermore, two of the hippocampus connections presented significant differences: right parahippocampal gyrus (*r* (280) = 0.13, *p* = 0.028) and the left medial temporal gyrus (*r* (280) = 0.14, *p* = 0.016). With regards to the descriptives (Supplemental Table S2a–c), POD participants generally present on average higher connectivity scores than no-POD (with exception of the amygdala and pallidum).

### Course connectivity analysis

In order to compare the course connectivity, ANOVAs of the difference scores (postoperative score minus preoperative score) were calculated. Herewith, the significant regions of the preoperative connectivities analyses constituted the basis for the subsequent analyses. With regard to the OFC, only the course connectivity in the right inferior frontal triangulate gyrus was significantly different between the groups (*F* (1, 176) = 4.47, *p* = 0.036). With respect to the descriptives, the group with POD displayed a greater change in connectivity than the group no-POD (no-POD *M* = 0.010, SD = 0.120 POD *M* = −0.052, SD = 0.122). The negative score indicates smaller postoperative measures compared to the preoperative measurements in POD patients. With regards to the ANOVA of the NAcc, the difference scores of connectivities with the right and left planum polare were significantly different between the groups (right: *F* (1, 176) = 9.03, *p* = 0.003; left: *F* (1, 176) = 3.99, *p* = 0.047). The descriptives show similar results as in the OFC, indicating a greater change in connectivity with smaller post measures in POD (right: no-POD *M* = 0.015, SD = 0.114; POD *M* = −0.068, SD = 0*.*112). Supplemental Table S3 displays the descriptives and results of each region from the course-connectivity analysis. As some distributions within the preoperative sample were non-normal, a non-parametric Wilcoxon test demonstrated similar significances with regards to these regions (with exception of the left planum polare). A matched sample comparison presented similar patterns and results (Supplemental Tables S4a–c, 5 and 6).

### Length of delirium

Based on the twice per day assessments of delirium, the average length of delirium of the 46 patients was 1.39 (half days) (SD = 1.26), with a maximum length of 5.5 (half days). By correlating the delirium duration with the single course connectivities no significant correlations were found (Supplemental Table S7). An exploratory analysis checking whether delirium duration and the parahippocampal course connectivity were independently associated with changes in memory parameter performances (i.e., PAL, SSP, TMT (A/B), VMR) presented also no significant differences.

### Postoperative connectivity analysis

**Fig. 3:**
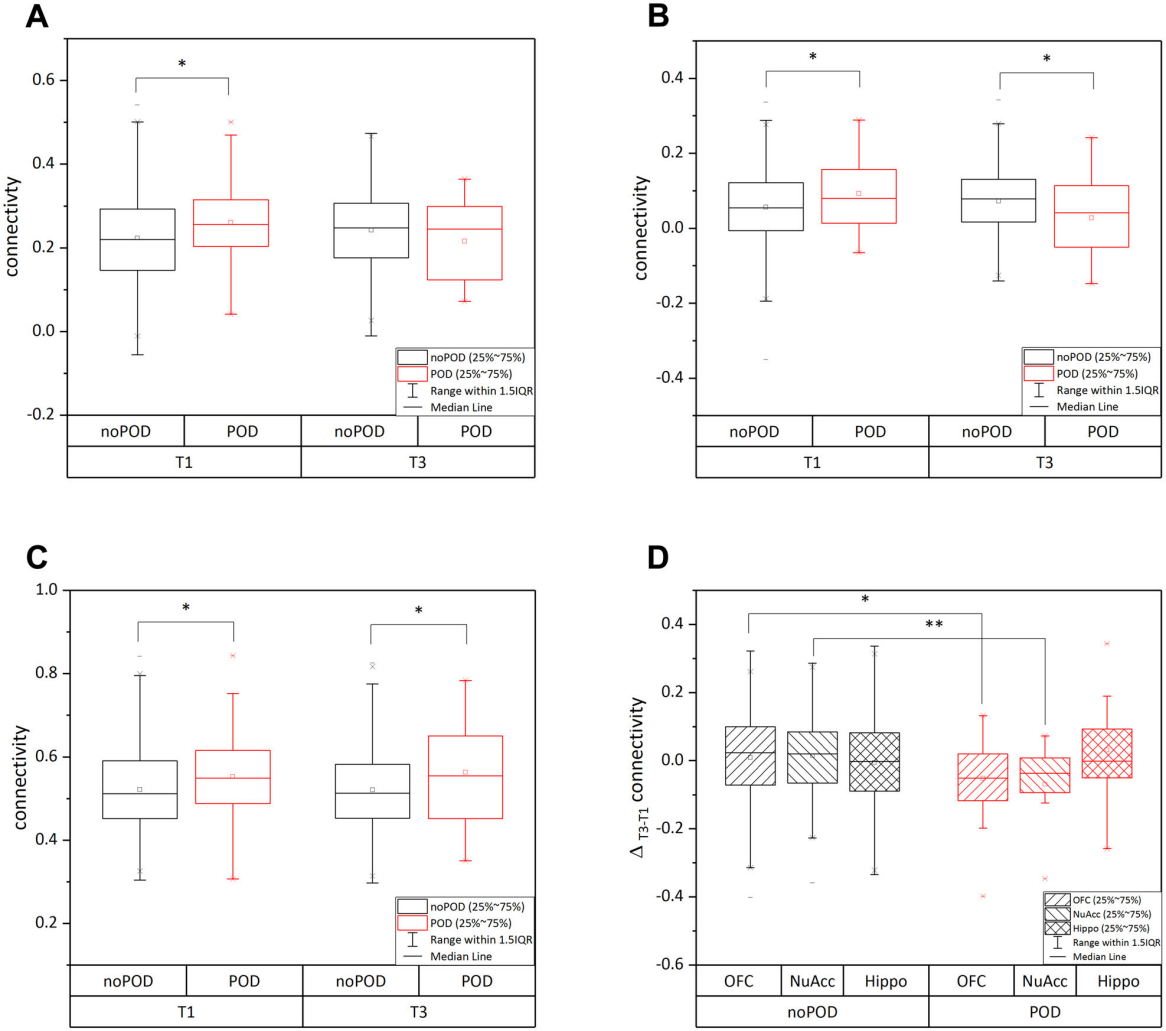
Pre-/post-operative scores (T1–T3). **A** Orbitofrontal cortex network: Right inferior frontal triangulate gyrus. **B** Nucleus accumbens network: Right planum polare. **C** Hippocampus network: Right hippocampus. **D** Significant course connectivities between no-POD and POD. Note: *p < 0.05. POD postoperative delirium.

The three connectivities showing statistically significant between-group differences in the course activity (i.e., right inferior frontal gyrus, bilateral planum polare) and for exploratory purposes also the hippocampus connectivities were analyzed. The right inferior frontal gyrus and the left planum polare presented no significant differences between the two groups (right inferior frontal gyrus: *F* (1, 176) = 1.27, *p* = 0.262; left planum polare: *F* (1, 176) = 2.38, *p* = 0.125). The right planum polare demonstrated a significant difference in postoperative connectivity between the two groups (*F* (1, 176) = 4.00, *p* = 0.047). Focusing on the hippocampal connectivities, the left parahippocampal gyrus (*F* (1, 188) = 3.99, *p* = 0.047), the right parahippocampal gyrus (*F* (1, 176) = 5.95, *p* = 0.016), and the left medial temporal gyrus (*F* (1, 176) = 4.18, *p* = 0.042) were significantly different. Supplemental Table S8 displays the average connectivities of the groups demonstrating lower average scores for patients with POD across the OFC and NAcc and higher scores within the hippocampus functional connectivities. Fig. 3(A–D) displays boxplots for three course connectivities (POD vs. no-POD).

## Discussion

The present study aimed to compare the transition of various ROIs with serial rs-fMRI assessments across patients exhibiting POD compared to no-POD patients to attain further insight into the pathology and course of delirium.

In preoperative rs-fMRI measurements, as hypothesized, we generally observed differences in functional connectivities, i.e., hyperconnectivity, in POD patients. Research of the group in South Korea^34,35^ who also collected preoperative rs-fMRI data in POD, demonstrating preoperative DLPFC ALFF changes among POD patients, did not report abnormal preoperative cortical connectivities, however, they did not address group x time interactions in detail given that their focus was on course changes. Our findings now demonstrate that POD patients already display increased functional connectivities (hyperconnectivity) preoperatively and not only during postoperative delirium as reported by Oh et al.^35^. The OFC, associated with decision-making and context-specific responding^58,59^, exhibited hyperconnectivities to the inferior temporal and inferior frontal triangulate gyrus, regions related to the verbal fluency, language processing, and response inhibition^60–63^. The NAcc, part of the ventral striatum and linked to reward and motivation^64–66^ demonstrated hyperconnections to the accumbens, Heschl gyrus, and planum polare, regions associated with auditory processing and auditory object/voice identification^67–70^. Interestingly, the connectivity aberrations in these ROIs occur bilaterally or on the right hemisphere. As the right hemisphere is proposed to be more involved with processing new information compared to the left hemisphere^71^ this would give reason for the coping issues of delirious participants in unfamiliar situations. Lastly, the hippocampus, a critical interface of the limbic system and memory processing, shows hyperconnectivities to regions influencing the recognition of environmental, social context, and facial stimuli next to spatial and episodic memory (i.e., parahippocampus, medial temporal gyrus, and hippocampus)^72–74^. However, an over-activation does not instantly imply effective use of neuronal resources. It may be the result of compensation mechanisms or an inefficient overuse of brain resources^75^. The deviation in connectivity could also be associated with imbalances in neurotransmitter levels. Dopamine excess appears to contribute to delirium development^10,48^, whereas a disproportion in dopamine levels due to disruptions in all three seed regions also positively correlate with positive symptoms and cognitive deficits of schizophrenia similar to delirium^47,76,77^. As the participants do not manifest phenotypically symptoms yet, the over-activation and neurotransmission may be within limits.

The course connectivity analysis revealed a trend for the OFC and NAcc connections to decrease as hypothesized among POD patients. More specifically, functional connectivties of the OFC to the right inferior frontal triangulate gyrus (i.e., response inhibition, executive function^63^) and the NAcc to the planum polare (i.e., auditory identification^69,70^) were different between the two groups. As research demonstrated age reductions in frontostriatal response, the decreases in connectivity may enhance this process and evolve into delirium^75^. Another explanation for a decrease could be a lack in cognitive reserve prior surgery as those with a high reserve cope better with network disruptions^78,79^. However, one common indicator for cognitive reserve, educational background^80^, did not present group differences, refuting this possible explanation. In line with prior research, subcortical connectivity appears to be sensitive to anesthesia, posing regions involved in delirium progression^31^. As opposed to prior research, the decrease in connectivity was not linked to the duration of delirium^22^. In our view, it appears to be conceivable that in a study with a higher number of severely ill POD patients with considerably longer delirium duration, accompanied with a higher incidence of neuroleptic intake, one might detect significant associations of, for instance, abnormal hippocampal course connectivities and impaired environmental recognition and memory encoding.

Postoperatively, the POD group differed with a decrease in connectivities from the NAcc to the right planum polare, and an increase in connectivities of the hippocampus to the parahippocampus and to the left medial temporal gyrus. Although not significantly different, the remaining connections within the OFC and NAcc indicate lower and within the hippocampus higher levels after delirium. Oh et al.^35^ suggested these reductions in functioning portray disconnections of striatal areas with their neurotransmitter origins. These disconnections possibly disturb the frontostriatal loop causing deficiency in attention, behavioral disinhibition, and decision-making. Contrary, the hippocampus functional connectivities may be more prone to respond with dysfunctional overuse, which may lead to memory deficits. Altogether, these findings propose disruptions in the underlying subcortical connectivities involved with the dopaminergic systems to influence upper-level cortical regions crucial for executive functioning evolving in symptoms of delirium.

Along with a decent sample size, providing greater statistical power, the study benefits of systematically analyzing pre-to post-operative changes among those developing delirium. Nevertheless, this study possesses limitations: The basis for the statistical analysis remained the significant preoperative connectivities leaving out the non-significant, which may also differ in the course analysis. Further, although the decent sample size, a greater and more equal sample size may consolidate and even enhance these findings. A lack of postoperative scans reduced the number for the course and post-analysis. Because of an inconsistency in daily delirium measurements (i.e., NuDesc), signs of delirium may have been bypassed. As ASA and surgical time constitute additional factors that may also have an impact independently of delirium, one should consider our results with caution. Additionally, although the difference in surgery site may portray a limitation, the study did not specifically recruit for this and left it to chance. Both the samples heterogeneity with respect to the site of surgery and ethnic homogeneity limit the generalizability. Further, expanding the sample with the Utrecht dataset was not possible because of heterogeneous technical issues (i.e., type of MRI scanner, scanner replacement). Moreover, since delirium was measured 3–5 months postoperatively and rs-fMRI connectivity measures can present moderate test-retest reliabilities, the appearance of the brain connectivity changes may be determined by this. A selection bias may have resulted with regards of the dropouts potentially even portraying worse conditions. As research illustrates a high association between cognitive deficits and delirium duration^22^ a larger sample including longer delirium would be beneficial in future studies. Lastly, we advise future research directions to also target the hippocampus specifically as our data analysis was not yet sufficient enough to answer the related questions satisfactorily. Past research demonstrated hippocampal hyperconnectivities and also parahippocampal atrophy to play a role in early disease stages of Alzheimer’s disease such as mild cognitive impairment^81–83^.

In conclusion, on the basis of serial rs-fMRI measures, we were able to demonstrate preoperative connectivity differences among those developing delirium compared to healthy non-delirious controls. Hyperconnectivties were found prior surgery across all three ROIs. In our course analyses, we could show that connectivities in the nucleus accumbens and orbitofrontal cortex decline, regions responsible for auditory identification and executive functioning. In line with prior studies, this study agrees with the notion that stable brain connectivities related to regions responsible for dopaminergic neurotransmission are required for healthy brain states.

## Supplementary information

Supplemental Tables

Supplemental Methods

Supplemental Figure 1

Supplemental Figure 2
