# Supplementary material for: Neurocognitive disorders in the elderly: altered functional resting-state hyperconnectivities in postoperative delirium patients
Source: Transl Psychiatry. 2021 Apr 12;11:213. doi: 10.1038/s41398-021-01304-y (PMC8041755; doi:10.1038/s41398-021-01304-y)
Supplement: Supplementary file 1 — Supplemental Tables [file 41398_2021_1304_MOESM1_ESM.docx]

| Preoperative | Parameter of interest | N  (no-POD/POD) | Patients without POD | Patients with POD | *H* value  (F value) | *p* value |
| --- | --- | --- | --- | --- | --- | --- |
| SSP  SSP  (log) | Span length | 234/45 | 5 (4/5) | 5 (4/5) | 1.54  1.54 | 0.214  0.214 |
| TMT-A  TMT-A  (log) | Time of completion (s) | 227/41 | 45 (36/60) | 55 (40/70) | 5.28  5.28 | *0.024*  *0.024* |
| TMT-B  TMT-B  (log)  TMT-B (ANOVA) | Time of completion (s) | 217/40 | 102 (86/138) | 117 (101/150) | 1.50  1.50  1.92 | 0.221  0.221  0.167 |
| VMR  (immediate)  VMR  (immediate) (log) | Number of correctly recognized items | 235/45 | 23 (22/24) | 23 (22/24) | 0.03  0.05 | 0.860  0.823 |
| VMR (delayed)  VMR (delayed)  (log) | Number of correctly recognized items after delay | 200/42 | 22 (20/23) | 22 (20/23) | 1.17  1.17 | 0.280  0.280 |
| PAL | Memory score | 233/44 | 13 (11/17) | 13 (10/16) | 1.12 | 0.291 |
| Median (25/75% percentiles); Abbreviations: POD, Post-operative Delirium; SSP, Spatial Span Length; TMT (A & B), Trail Making Test; VRM (immediate & delayed), Verbal Recognition Memory; PAL, Paired Associate Learning; SRT, Simple Reaction Test. | | | | | | |

**Supplemental Table S1a:** Preoperative differences in neurocognitive parameters (CANTAB)

**Supplemental Table S1b:** Postoperative differences in neurocognitive parameters (CANTAB)

| Postoperative | Parameter of interest | N  (no-POD/POD) | Patients without POD | Patients with POD | *H* value  (F value) | *p* value |
| --- | --- | --- | --- | --- | --- | --- |
| SSP  SSP  (log) | Span length | 156/19 | 5 (5/5) | 5 (5/5) | 0.41  0.36 | 0.521  0.551 |
| TMT-A  TMT-A  (log) | Time of completion (s) | 154/19 | 42 (33/56) | 47 (40/60) | 1.43  1.23 | 0.232  0.268 |
| TMT-B  TMT-B  (log) | Time of completion (s) | 150/18 | 98 (78/124) | 109 (97/131) | 1.93  1.93 | 0.165  0.165 |
| VRM (immediate)  VRM  (immediate) (log) | Number of correctly recognized items | 156/19 | 23 (22/24) | 22 (20/23) | 2.43  2.62 | 0.119  0.106 |
| VMR  (delayed)  VMR  (delayed)  (log) | Number of correctly recognized items after delay | 152/19 | 22 (20/23) | 22 (21/22) | 0.03  0.03 | 0.856  0.856 |
| PAL | Memory score | 153/18 | 15 (12/18) | 14 (12/18) | 0.11 | 0.737 |
| Median (25/75% percentiles); Abbreviations: POD, Post-operative Delirium; SSP, Spatial Span Length; TMT (A & B), Trail Making Test; VRM (immediate & delayed), Verbal Recognition Memory; PAL, Paired Associate Learning | | | | | | |

**Supplemental Table S2a**. Descriptives of preoperative connectivity analysis of the orbitofrontal cortex network

| Preoperative connectivity | No-POD group  (n = 237) | POD group  (n = 46) | *R* value | *p* value |
| --- | --- | --- | --- | --- |
| Medial Frontal Cortex | 0.29 (0.14) | 0.32 (0.16) | 0.08 | 0.203 |
| Left medial temporal gyrus, posterior division | 0.20 (0.11) | 0.22 (0.13) | 0.07 | 0.242 |
| Right medial temporal gyrus, posterior division | 0.18 (0.10) | 0.21 (0.14) | 0.12 | 0.053 |
| Left accumbens | 0.18 (0.12) | 0.18 (0.15) | -0.00 | 0.970 |
| Right accumbens | 0.19 (0.12) | 0.18 (0.15) | -0.05 | 0.421 |
| Subcallosal gyrus | 0.24 (0.11) | 0.27 (0.13) | 0.09 | 0.133 |
| Left frontal operculum cortex | 0.24 (0.11) | 0.23 (0.11) | -0.02 | 0.731 |
| Right frontal operculum cortex | 0.19 (0.12) | 0.19 (0.12) | -0.01 | 0.869 |
| Left inferior frontal triangulate gyrus | 0.21 (0.11) | 0.21 (0.11) | 0.02 | 0.693 |
| Right inferior frontal triangulate gyrus | 0.22 (0. 11) | 0.26 (0.10) | 0.14 | *0.021* |
| Left inferior temporal gyrus, anterior division | 0.17 (0.12) | 0.18 (0.10) | 0.02 | 0.797 |
| Right inferior temporal gyrus, anterior division | 0.16 (0.11) | 0.20 (0.11) | 0.12 | *0.037* |
| Left planum polare | 0.15 (0.11) | 0.18 (0.11) | 0.11 | 0.073 |
| Right planum polare | 0.16 (0.11) | 0.18 (0.13) | 0.08 | 0.189 |
| Left inferior colliculus | 0.16 (0.09) | 0.17 (0.11) | 0.06 | 0.297 |
| Right inferior colliculus | 0.15 (0.09) | 0.17 (0.11) | 0.08 | 0.158 |
| Sex as covariate; Continuous variables expressed as mean (standard deviation); Abbreviations: POD, Postoperative Delirium. | | | | |

**Supplemental Table S2b.** Descriptives of the preoperative connectivity analysis of the nucleus accumbens

| Preoperative connectivity | No-POD group  (n = 237) | POD group  (n = 46) | *R* value | *p* value |
| --- | --- | --- | --- | --- |
| Left accumbens | 0.73 (0.13) | 0.80 (0.11) | 0.20 | *0.001* |
| Right accumbens | 0.74 (0.13) | 0.80 (0.14) | 0.17 | *0.004* |
| Left paracingulate gyrus | 0.13 (0.07) | 0.13 (0.09) | -0.03 | 0.625 |
| Right paracingulate gyrus | 0.12 (0.07) | 0.11 (0.08) | -0.02 | 0.749 |
| Left caudate nucleus | 0.24 (0.10) | 0.26 (0.10) | 0.08 | 0.162 |
| Right caudate nucleus | 0.23 (0.10) | 0.25 (0.11) | 0.07 | 0.264 |
| Left putamen | 0.15 (0.09) | 0.14 (0.09) | -0.04 | 0.514 |
| Right putamen | 0.16 (0.09) | 0.15 (0.09) | -0.03 | 0.649 |
| Medial frontal cortex | 0.12 (0.09) | 0.11 (0.14) | -0.06 | 0.295 |
| Subcallosal gyrus | 0.17 (0.10) | 0.19 (0.13) | 0.06 | 0.352 |
| Left frontal orbital cortex | 0.10 (0.07) | 0.10 (0.09) | 0.01 | 0.919 |
| Right frontal orbital cortex | 0.10 (0.07) | 0.10 (0.09) | 0.03 | 0.638 |
| Left heschl’s gyrus | 0.06 (0.10) | 0.08 (0.11) | 0.07 | 0.267 |
| Right heschl’s gyrus | 0.05 (0.11) | 0.10 (0.10) | 0.16 | *0.007* |
| Left planum polare | 0.06 (0.09) | 0.09 (0.09) | 0.13 | *0.026* |
| Right planum polare | 0.06 (0.10) | 0.09 (0.09) | 0.14 | *0.018* |
| Left amygdala | 0.07 (0.09) | 0.05 (0.08) | -0.74 | 0.214 |
| Right amygdala | 0.06 (0.09) | 0.05 (0.11) | -0.52 | 0.383 |
| Left pallidum | 0.10 (0.09) | 0.09 (0.09) | -0.05 | 0.425 |
| Right pallidum | 0.10 (0.09) | 0.08 (0.09) | -0.07 | 0.242 |
| Sex as covariate; Continuous variables expressed as mean (standard deviation); Abbreviations: POD, Postoperative Delirium. | | | | |

**Supplemental Table S2c**. Descriptives of preoperative connectivity analysis of the hippocampus network

| Preoperative connectivity | No-POD group  (n = 237) | POD group  (n = 46) | *R* value | *p* value |
| --- | --- | --- | --- | --- |
| Medial frontal cortex | 0.21 (0.13) | 0.22 (0.11) | 0.04 | 0.537 |
| Left amygdala | 0.34 (0.14) | 0.35 (0.13) | 0.05 | 0.402 |
| Right amygdala | 0.35 (0.12) | 0.37 (0.13) | 0.09 | 0.151 |
| Left parahippocampal gyrus, anterior division | 0.27 (0.11) | 0.29 (0.11) | 0.07 | 0.234 |
| Right parahippocampal gyrus, anterior division | 0.27 (0.11) | 0.28 (0.12) | 0.02 | 0.710 |
| Left parahippocampal gyrus, posterior division | 0.32 (0.09) | 0.34 (0.09) | 0.10 | 0.084 |
| Right parahippocampal gyrus, posterior division | 0.34 (0.10) | 0.38 (0.10) | 0.13 | *0.028* |
| Left superior temporal gyrus, anterior division | 0.17 (0.13) | 0.18 (0.13) | 0.06 | 0.281 |
| Right superior temporal gyrus, anterior division | 0.18 (0.11) | 0.20 (0.12) | 0.10 | 0.096 |
| Left medial temporal gyrus, anterior division | 0.18 (0.12) | 0.23 (0.14) | 0.14 | *0.016* |
| Right medial temporal gyrus, anterior division | 0.19 (0.11) | 0.21 (0.12) | 0.05 | 0.377 |
| Left temporal pole | 0.17 (0.09) | 0.18 (0.10) | 0.08 | 0.206 |
| Right temporal pole | 0.17 (0.09) | 0.18 (0.09) | 0.08 | 0.175 |
| Left hippocampus | 0.50 (0.10) | 0.53 (0.11) | 0.11 | 0.055 |
| Right hippocampus | 0.52 (0.10) | 0.55 (0.11) | 0.12 | 0.054 |
| Left thalamus | 0.08 (0.08) | 0.09 (0.09) | 0.04 | 0.467 |
| Right thalamus | 0.07 (0.09) | 0.08 (0.09) | 0.06 | 0.348 |
| Sex as covariate; Continuous variables expressed as mean (standard deviation); Abbreviations: POD, Postoperative Delirium. | | | | |

**Supplemental Table S3.** Descriptives of course connectivity analysis

| Course connectivity | No-POD group (n = 159) | POD group  (n = 19) | *F* value | *p* value |
| --- | --- | --- | --- | --- |
| Orbitofrontal cortex network  Right medial temporal gyrus, posterior division  Right inferior frontal triangulate gyrus  Right inferior temporal gyrus, anterior division | 0.02 (0.12)  0.01 (0.12)  0.01 (0.14) | -0.02 (0.11)  -0.05 (0.12)  -0.02 (0.13) | 1.91  4.47  0.71 | 0.169  *0.036*  0.401 |
| Nucleus accumbens network  Left accumbens  Right accumbens  Right heschl’s gyrus  Left planum polare  Right planum polare | 0.01 (0.14)   - 1. (0.14)   2. (0.19)   3. (0.11)   0.02 (0.11) | -0.03 (0.17)  -0.02 (0.18)  -0.04 (0.14)  -0.05 (0.13)  -0.07 (0.11) | 1.10  0.72  2.67  3.99  9.03 | 0.295  0.398  0.104  *0.047*  *0.003* |
| Hippocampus Network  Left parahippocampal gyrus, posterior division  Right parahippocampal gyrus, posterior division  Left medial temporal gyrus, anterior division  Left hippocampus  Right hippocampus | 0.01 (0.11)   - 1. (0.12)   -0.02 (0.13)  -0.00 (0.12)  -0.01 (0.12) | 0.03 (0.15)  0.04 (0.17)   - 1. (0.12)   2. (0.14)   0.03 (0.14) | 0.44  1.42  0.69  0.61  1.62 | 0.507  0.243  0.407  0.434  0.209 |
| Course connectivity presents the difference score of postoperative and preoperative scores; Continuous variables expressed as mean (standard deviation); Abbreviations: POD, postoperative delirium. | | | | |

**Supplemental Table S4a**. Descriptives of preoperative connectivity analysis of the orbitofrontal cortex network: Matched sample

| Preoperative connectivity | No-POD group  (n = 46) | POD group  (n = 46) | *R* value | *p* value |
| --- | --- | --- | --- | --- |
| Medial Frontal Cortex | 0.30 (0.16) | 0.32 (0.16) | 0.08 | 0.461 |
| Left medial temporal gyrus, posterior division | 0.19 (0.11) | 0.22 (0.13) | 0.18 | 0.265 |
| Right medial temporal gyrus, posterior division | 0.16 (0.11) | 0.21 (0.14) | 0.21 | *0.041* |
| Left accumbens | 0.20 (0.11) | 0.18 (0.15) | -0.07 | 0.520 |
| Right accumbens | 0.21 (0.11) | 0.18 (0.15) | -0.11 | 0.282 |
| Subcallosal gyrus | 0.24 (0.12) | 0.27 (0.13) | 0.14 | 0.196 |
| Left frontal operculum cortex | 0.24 (0.12) | 0.23 (0.11) | -0.07 | 0.524 |
| Right frontal operculum cortex | 0.20 (0.13) | 0.19 (0.12) | -0.05 | 0.627 |
| Left inferior frontal triangulate gyrus | 0.20 (0.11) | 0.21 (0.11) | 0.05 | 0.637 |
| Right inferior frontal triangulate gyrus | 0.22 (0.12) | 0.26 (0.10) | 0.18 | 0.090 |
| Left inferior temporal gyrus, anterior division | 0.17 (0.11) | 0.18 (0.10) | 0.04 | 0.685 |
| Right inferior temporal gyrus, anterior division | 0.13 (0.10) | 0.20 (0.11) | 0.30 | *0.004* |
| Left planum polare | 0.16 (0.10) | 0.18 (0.11) | 0.11 | 0.288 |
| Right planum polare | 0.17 (0.10) | 0.18 (0.13) | 0.05 | 0.616 |
| Left inferior colliculus | 0.16 (0.09) | 0.17 (0.11) | 0.07 | 0.504 |
| Right inferior colliculus | 0.16 (0.10) | 0.17 (0.11) | 0.03 | 0.808 |
| Sex as covariate; Continuous variables expressed as mean (standard deviation); Abbreviations: POD, Postoperative Delirium. | | | | |

**Supplemental Table S4b.** Descriptives of preoperative connectivity analysis of the nucleus accumbens network: Matched sample

| Preoperative connectivity | No-POD group  (n = 46) | POD group  (n = 46) | *R* value | *p* value |
| --- | --- | --- | --- | --- |
| Left accumbens | 0.73 (0.14) | 0.80 (0.11) | 0.26 | *0.012* |
| Right accumbens | 0.74 (0.13) | 0.80 (0.14) | 0.23 | *0.028* |
| Left paracingulate gyrus | 0.14 (0.08) | 0.13 (0.09) | -0.09 | 0.392 |
| Right paracingulate gyrus | 0.12 (0.77) | 0.11 (0.08) | -0.07 | 0.525 |
| Left caudate nucleus | 0.24 (0.09) | 0.26 (0.10) | 0.11 | 0.296 |
| Right caudate nucleus | 0.26 (0.09) | 0.25 (0.11) | 0.04 | 0.705 |
| Left putamen | 0.17 (0.07) | 0.14 (0.09) | -0.15 | 0.145 |
| Right putamen | 0.18 (0.08) | 0.15 (0.09) | -0.14 | 0.170 |
| Medial frontal cortex | 0.12 (0.10) | 0.11 (0.14) | -0.04 | 0.699 |
| Subcallosal gyrus | 0.18 (0.09) | 0.19 (0.13) | 0.04 | 0.731 |
| Left frontal orbital cortex | 0.11 (0.06) | 0.10 (0.09) | -0.07 | 0.485 |
| Right frontal orbital cortex | 0.10 (0.06) | 0.10 (0.09) | 0.02 | 0.850 |
| Left heschl’s gyrus | 0.07 (0.11) | 0.08 (0.11) | 0.06 | 0.598 |
| Right heschl’s gyrus | 0.08 (0.10) | 0.10 (0.10) | 0.12 | 0.261 |
| Left planum polare | 0.07 (0.08) | 0.09 (0.09) | 0.17 | 0.116 |
| Right planum polare | 0.05 (0.08) | 0.09 (0.09) | 0.24 | *0.022* |
| Left amygdala | 0.08 (0.07) | 0.05 (0.08) | -0.19 | 0.077 |
| Right amygdala | 0.05 (0.07) | 0.05 (0.11) | -0.02 | 0.835 |
| Left pallidum | 0.13 (0.07) | 0.09 (0.09) | -0.21 | *0.044* |
| Right pallidum | 0.12 (0.09) | 0.08 (0.09) | -0.20 | 0.060 |
| Sex as covariate; Continuous variables expressed as mean (standard deviation); Abbreviations: POD, Postoperative Delirium. | | | | |

**Supplemental Table S4c.** Descriptives of preoperative connectivity analysis of the hippocampus network: Matched sample

| Preoperative connectivity | No-POD group  (n = 46) | POD group  (n = 46) | *R* value | *p* value |
| --- | --- | --- | --- | --- |
| Medial frontal cortex | 0.19 (0.10) | 0.22 (0.11) | 0.14 | 0.192 |
| Left amygdala | 0.33 (0.13) | 0.35 (0.13) | 0.09 | 0.384 |
| Right amygdala | 0.34 (0.12) | 0.37 (0.13) | 0.13 | 0.232 |
| Left parahippocampal gyrus, anterior division | 0.28 (0.11) | 0.29 (0.11) | 0.07 | 0.504 |
| Right parahippocampal gyrus, anterior division | 0.27 (0.11) | 0.28 (0.12) | 0.01 | 0.934 |
| Left parahippocampal gyrus, posterior division | 0.31 (0.09) | 0.34 (0.09) | 0.21 | *0.050* |
| Right parahippocampal gyrus, posterior division | 0.33 (0.10) | 0.38 (0.10) | 0.21 | *0.045* |
| Left superior temporal gyrus, anterior division | 0.16 (0.14) | 0.18 (0.13) | 0.09 | 0.405 |
| Right superior temporal gyrus, anterior division | 0.16 (0.11) | 0.20 (0.12) | 0.21 | *0.050* |
| Left medial temporal gyrus, anterior division | 0.16 (0.11) | 0.23 (0.14) | 0.25 | *0.015* |
| Right medial temporal gyrus, anterior division | 0.16 (0.09) | 0.21 (0.12) | 0.22 | *0.035* |
| Left temporal pole | 0.15 (0.08) | 0.18 (0.10) | 0.19 | 0.090 |
| Right temporal pole | 0.15 (0.09) | 0.18 (0.09) | 0.21 | *0.048* |
| Left hippocampus | 0.49 (0.10) | 0.53 (0.11) | 0.21 | *0.043* |
| Right hippocampus | 0.51 (0.09) | 0.55 (0.11) | 0.25 | *0.019* |
| Left thalamus | 0.07 (0.08) | 0.09 (0.09) | 0.12 | 0.264 |
| Right thalamus | 0.07 (0.09) | 0.84 (0.08) | 0.10 | 0.364 |
| Sex as covariate; Continuous variables expressed as mean (standard deviation); Abbreviations: POD, Postoperative Delirium. | | | | |

**Supplemental Table S5.** Descriptives of course connectivity analysis: Matched sample

| Course connectivity | No-POD group (n = 29) | POD group  (n = 19) | *F* value | *p* value |
| --- | --- | --- | --- | --- |
| Orbitofrontal cortex network  Right medial temporal gyrus, posterior division  Right inferior frontal triangulate gyrus  Right inferior temporal gyrus, anterior division | 0.04 (0.13)  0.03 (0.15)  0.07 (0.14) | -0.02 (0.11)  -0.51 (0.13)  -0.02 (0.13) | 2.40  3.63  4.16 | 0.128  0.063  *0.047* |
| Nucleus accumbens network  Left accumbens  Right accumbens  Right heschl’s gyrus  Left planum polare  Right planum polare | -0.01 (0.15)  -0.00 (0.15)  0.01 (0.12)  0.00 (0.12)  0.03 (0.10) | -0.03 (0.17)  -0.02 (0.18)  -0.04 (0.14)  -0.05 (0.13)  -0.06 (0.11) | 0.15  0.16  1.46  1.94  9.87 | 0.702  0.693  0.234  0.170  *0.003* |
| Hippocampus Network  Left parahippocampal gyrus, posterior division  Right parahippocampal gyrus, posterior division  Left medial temporal gyrus, anterior division  Left hippocampus  Right hippocampus | 0.03 (0.12)   - 1. (0.14)   0.02 (0.14)  0.00 (0.12)  0.02 (0.12) | 0.04 (0.15)  0.04 (0.17)  0.00 (0.12)    0.02 (0.14)  0.03 (0.14) | 0.00  0.22  0.05  0.33  0.15 | 0.950  0.640  0.830  0.570  0.696 |
| Course connectivity presents the difference score of post-operative and pre-operative scores; Continuous variables expressed as mean (standard deviation); Abbreviations: POD, post-operative delirium. | | | | |

**Supplemental Table S6.** Descriptives of post-operative connectivity analysis: Matched sample

| Post-operative connectivity | No-POD group (n = 29) | POD group  (n = 19) | *F* value | *p* value |
| --- | --- | --- | --- | --- |
| Orbitofrontal cortex network  Right medial temporal gyrus, posterior division  Right inferior frontal triangulate gyrus  Right inferior temporal gyrus, anterior division | 0.19 (0.11)  0.26 (0.13)  0.20 (0.11) | 0.23 (0.13)  0.22 (0.09)  0.19 (0.13) | 0.99  1.37  0.05 | 0.324  0.249  0.834 |
| Nucleus accumbens network  Left accumbens  Right accumbens  Right heschl’s gyrus  Left planum polare  Right planum polare | 0.71 (0.13)  0.73 (0.15)  0.07 (0.10)  0.07 (0.11)  0.07 (0.10) | 0.78 (0.16)  0.80 (0.14)  0.06 (0.11)   - 1. (0.12)   0.03 (0.10) | 2.83  2.69  0.01  1.00  2.32 | 0.100  0.108  0.912  0.323  0.134 |
| Hippocampus network  Left parahippocampal gyrus, posterior division  Right parahippocampal gyrus, posterior division  Left medial temporal gyrus, anterior division  Left hippocampus  Right hippocampus | 0.33 (0.10)  0.37 (0.10)  0.19 (0.13)  0.49 (0.09)  0.53 (0.09) | 0.38 (0.12)  0.41 (0.10)  0.22 (0.12)  0.53 (0.11)  0.56 (0.11) | 2.23  2.20  0.68  2.06  1.21 | 0.142  0.145  0.413  0.158  0.278 |
| Continuous variables expressed as mean (standard deviation); Abbreviations: POD, Post-operative Delirium. | | | | |

**Supplemental Table S7.** The association between length of delirium and regions of course connectivity analysis

| Length of delirium & course connectivity | *R* | *R^2^* | *F* value | *p* value |
| --- | --- | --- | --- | --- |
| Orbitofrontal cortex network  Right medial temporal gyrus, posterior division  Right inferior frontal triangulate gyrus  Right inferior temporal gyrus, anterior division | 0.05  0.04  0.43 | 0.003  0.002  0.183 | 0.05  0.03  3.81 | 0.835  0.861  0.068 |
| Nucleus accumbens network  Left accumbens  Right accumbens  Right heschl’s gyrus  Left planum polare  Right planum polare | 0.03  0.09  0.28  0.06  0.33 | 0.001  0.008  0.081  0.004  0.107 | 0.01  0.13  1.50  0.06  2.03 | 0.913  0.723  0.238  0.807  0.172 |
| Hippocampus network  Left parahippocampal gyrus, posterior division  Right parahippocampal gyrus, posterior division  Left medial temporal gyrus, anterior division  Left hippocampus  Right hippocampus | 0.45  0.12  0.20  0.29  0.19 | 0.204  0.015  0.038  0.082  0.083 | 4.35  0.27  0.68  1.53  0.67 | 0.052  0.613  0.422  0.233  0.425 |
| Calculated from the 19 patients with postoperative delirium taken into account in the course connectivity analysis. | | | | |

**Supplemental Table S8.** Descriptives of postoperative connectivity analysis

| Postoperative connectivity | No-POD group (n = 159) | POD group  (n = 19) | *F* value | *p* value |
| --- | --- | --- | --- | --- |
| Orbitofrontal cortex network  Right inferior frontal triangulate gyrus | 0.24 (0.01) | 0.22 (0.09) | 1.27 | 0.262 |
| Nucleus accumbens network  Left planum polare  Right planum polare | 0.07 (0.10)  0.07 (0.09) | - 1. (0.12)   0.03 (0.10) | 2.38  4.00 | 0.125  *0.047* |
| Hippocampus network  Left parahippocampal gyrus, posterior division  Right parahippocampal gyrus, posterior division  Left medial temporal gyrus, anterior division  Left hippocampus  Right hippocampus | 0.33 (0.10)  0.35 (0.10)  0.17 (0.11)  0.50 (0.10)  0.52 (0.10) | 0.38 (0.12)  0.41 (0.10)  0.23 (0.12)  0.53 (0.11)  0.56 (0.11) | 3.99  5.95  4.18  1.78  3.08 | *0.047*  *0.016*  *0.042*  0.184  0.081 |
| Continuous variables expressed as mean (standard deviation); Abbreviations: POD, Postoperative Delirium. | | | | |
