# Supplementary material for: Neurocognitive disorders in the elderly: altered functional resting-state hyperconnectivities in postoperative delirium patients
Source: Transl Psychiatry. 2021 Apr 12;11:213. doi: 10.1038/s41398-021-01304-y (PMC8041755; doi:10.1038/s41398-021-01304-y)
Supplement: Supplementary file 2 — Supplemental Methods [file 41398_2021_1304_MOESM2_ESM.docx]

Supplement Methods

Study procedure:

The study was conducted by physicians and researchers with data collection performed by a trained and supervised research team. Clinical data were recorded in case report forms. Clinical, neuropsychological and neuroimaging data were filled in a local data management system. After 400 patients were included, a planned interim analysis of pre-operative data was conducted for a parameter reduction the final analysis. The next final analysis was done after study completion.

Postoperative Delirium

Delirium screening was started in the post- anesthesia recovery room and repeated twice per day at 8:00am and 7:00pm (±1h) up to seven days after surgery. Either study physicians, study nurses and study assistants under supervision of a study physician visited. Patients without further information on POD incidence were sorted out. Delirium incidence was according to the following questionnaire criteria present: 2 cumulative points on the Nursing Delirium Screening Scale (Nu-DESC) and/or a positive Confusion Assessment Method (CAM) score and/or a positive CAM for the Intensive Care Unit (CAM-ICU) and/or patient chart review that shows descriptions of delirium (e.g. confused, agitated, drowsy, disorientated, delirious, received antipsychotic therapy).

Non-surgical control group: In order to adjust for natural learning effects in cognitive testing, 114 patients underwent the same inclusion/exclusion criteria but did not indicate the need for surgery were invited to perform neuropsychological testing at baseline as well as after one week and three months. Non-surgical control participants were recruited from outpatient clinics, primary care, elderly homes and via public talks calls.

Secondary outcomes

Apart from postoperative delirium, pre-and post-operative cognitive impairment and length of hospital stay, the following variables were considered eligible as independent variables in the analysis: age, MMSE, ASA-PS, Charlson comorbidity index, coronary artery disease, preoperative tumor, lymphoma or leukaemia, longterm medication with benzodiazepines, anticholinergic medication on postoperative day one, functional impairment measured in the Barthel Index or Instrumental Activities of Daily Living, performance in the Grooved Pegboard Test (time for completion), Simple Span length, free recall performance in the Verbal Recognition Memory test, memory score in the Paired Associate Learning test, Simple Reaction Time (mean latency for correct trials), Trail-Making-Test B (time for completion), frailty, malnutrition, duration of anaesthesia and surgery, type of anaesthesia, site of surgery, duration of stay at the intensive care unit, postoperative pain, preoperative hemoglobin, tryptophan in peripheral blood, IL6, troponin T, low- and high density lipoprotein concentration, total blood cholesterol concentration, zonulin, γ-glutamyltransferase activity, albumin concentration, global brain volume, cortical thickness in the posterior cingulate cortex, isthmus cinguli, insula, precentral gyrus, lingula, rostral and caudal middle frontal gyrus, lateral orbitofrontal cortex, pars orbitalis and opercularis, midlle temporal gyrus, volume of the posterior cingulate cortex, inferior parietal cortex, insula, precentral gyrus, pars orbitalis and triangularis, superior temporal gyrus, fusiform gyrus and entorhinal cortex, ncl. accumbens, putamen, thalamus, hippocampus, brain stem, cerebellar cortex, right superior longitudinal fascicle and temporal portion of the left superior longitudinal fascicle, mean curvature of the cuneus and precuneus, superior temporal sulcus, inferior temporal and the fusiform gyrus, fractional anistropy of the anterior thalamic radiation, left cingulate gyrus, forceps major and minor, right superior longitudinal fascicle, right uncinate fascicle, mean diffusivity of the anterior thalamic radiation, forceps major and minor, right superior longitudinal fascicle and right uncinate fascicle as well as mean kurtosis of water diffusion in the left anterior thalamic radiation, left corticospinal tract, left inferior fronto-occipital fascicle and left uncinate fascicle.

**Loss to follow-up**

Internal notes, derived from contact with relatives, patient files and resident registration offices data were used to make a follow-up status of the patients. In case of post-operative completion of the neuropsychological testing, the patient was allocated to the successful follow-up group. Others, such as death prior invitation were determined as dead before follow-up. The first analysis of loss to follow-up, the patients who have been invited were compared to those deceased, despite whether they completed the neuropsychological testing. Those patients that merely completed the questionnaires but not the neurocognitive assessment were also included in the successful follow-up on the basis of their willingness to participate in future assessments. With regards to the second analysis, these patients were then treated as lack of compliance. When patients did not return for follow-up measures the exact date of death was obtained and not proven to be alive at 90 days post-operatively by referring medical records and personal contact.

The exact date of death was obtained for all patients who did not return for the follow-up assessment and could not be proven to be alive at 90 days after surgery from the medical records or due to personal contact. For this purpose, we obtained the exact date of deceasing from the patient files or the resident’s registration office. 90-days mortality was only obtained for patients recruited in Berlin.

# Data collection and clinical assessment

A structured patient interview and clinical examination for demographic and clinical data was assessed by questionnaires and the patient’s case file. In case of need, additional medical reports were obtained from the patients. The study was conducted by physicians and researchers with data collection performed by a trained and supervised research team. Data were entered in the electronic clinical case report form (eCRF) SecuTrial® (interActive Systems, Berlin, Germany).

### Determination of sample size total study

The BioCog project design gave the possibility to perform multivariate risk prediction derived from stepwise logistic regression analysis and machine learning. With a 2-step strategy one referred to a training (exploratory) and a test (validation) data set. Whereas the training set defined models, the test set indicated the models prediction accuracy. As the number of predictors exceeds 100 among this large dataset, the models are prone to over-fitting. Therefore, model performance can be tested in an independent sample. Generally, one assumed up to 30% to develop postoperative delirium and up to 50% postoperative neurocognitive disorder (1). As POD/POCD studies with participant samples under 100 at the point of study design construction did not refer to effect sizes, one referred to effect sizes of greater imaging studies on Alzheimer’s dementia (Hedge’s g = 0.5-1.5). While assuming that up to 30% will indicate POD, 10% will drop-out and an Hedge’s of 0.5, the training set sample size of 200 participants will achieve 80% power (two-sided α=0.05). With regards to POCD, a training set of 400 participants shall be sufficient. According to previous studies effect sizes, 800 patients should be sufficient for an independent test-sample leading to a total of 1200 patients.

For the determination of the sample size of this study please refer to the flowchart in the supplemental figure 2.

# Sociodemographic data

Educational status was assessed with the WHO 1997 International Standard Classification of Education (ISCED 1997). ISCED levels were grouped in five categories referring to level 1 to 5. The ASA Physical Status scale was not dichotomized into multiple groups as conducted within Lachmann et al. (2019) (2).

# Perioperative parameters

Each visit (up to seven days) postoperatively pain scores were assessed with the Non-Visual Rating Scale (NRS), Behavioral Pain Scale (BPS) and the Critical-Care Pain Observation Tool (CPOT). One positive score was sufficient for pain definition (NRS≥5/10, BPS≥6/12, CPOT≥3/8). Pain was not significantly different between the two groups ($X^{2}$ = 2.488, *p* = .115). Surgery duration was determined by the electronic patient file. Site of surgery was mainly divided into intracranial/intrathoraric, abdominal or pelic/peripheral. Subdivisions can be made into general surgery (n = 65), opthalamic surgery (n = 20), dermatology (n = 1), gynecology ( n = 31), cardiac surgery (n = 1), otorhinolaryngynology (n = 23), maxillogacial surgery (n = 13), neurosurgery (n = 28), trauma and orthopedic surgery (n = 58) and urology (n = 43).

**Neuropsychological data**

PAL first trial memory score: Boxes were randomly displayed on the screen and presented one at a time. Within boxes patterns in the middle of the screen are shown, whereas the participant has to touch the box where the pattern was presented. Participants had up to ten attempts or trials. In case of errors a representation of the pattern was performed in order to remind the participant. In case of success the participant moved to the next stage, in case of too much errors or stage incompletion the test terminated. First trial memory score was the number of patterns correctly located after the first trial, summed across the stages completed (range 0-26 in the clinical mode, with 26 meaning all the patterns were correctly located for all stages first time).

VRM immediate/delayed recognition: A list of 12 words was presented the participant once and immediately asked to recall as many words they remember. After twenty minutes participants were presented a twenty-four-word list of which they had to identify the words from before (with 12 false distractors).

TMT-A/B: With regards to this paper-pencil task, participants had to connect 25 targets in a specific sequence. The first (A) part requested participants to connect numbers (1,2,3, etc.) whereas the second part (B) requested them to connect numbers and letters in alternating order (1, A, 2, B, etc.). The test instructor corrected participants in case of error. Completion times served in order to examine cognitive functions such as executive function.

SSP: Participants have to remember in the spatial span length task the order of the changing color of presented white square boxes. How good a participant remembers the order the better the visualspatial working memory capacity is.

Missing Data:

When data were incomplete with regards to the sociodemographic, the peri-operative and neuropsychological variables values were imputed and declared as missing values.

Two research assessors independently conducted a plausibility check for the data (including open-text entries). Missing values were imputed in case of incompletion. In case a value is missing and the test administer reported an impairment of concentration or poor understanding of the instructions, the worst performance value was imputed. Random missing values due to technical difficulties or environmental disturbances forest imputation was used. No imputation was performed when neuropsychological testing data were completely missing. Imputations were conducted with the missForest package for R Statistical Software.

References

1. Winterer G, Androsova G, Bender O, et al. Personalized risk prediction of postoperative cognitive impairment – rationale for the EU-funded BioCog project. European Psychiatry 2018;50:34–9.

2. Lachmann, G., Kant, I., Lammers, F., Windmann, V., Spies, C., Speidel, S., ... & de Bresser, J. (2019). Cerebral microbleeds are not associated with postoperative delirium and postoperative cognitive dysfunction in older individuals. *PloS one*, *14*(6), e0218411.
