# Supplementary figures and images for: Neurocognitive disorders in the elderly: altered functional resting-state hyperconnectivities in postoperative delirium patients
Source: Transl Psychiatry. 2021 Apr 12;11:213. doi: 10.1038/s41398-021-01304-y (PMC8041755; doi:10.1038/s41398-021-01304-y)

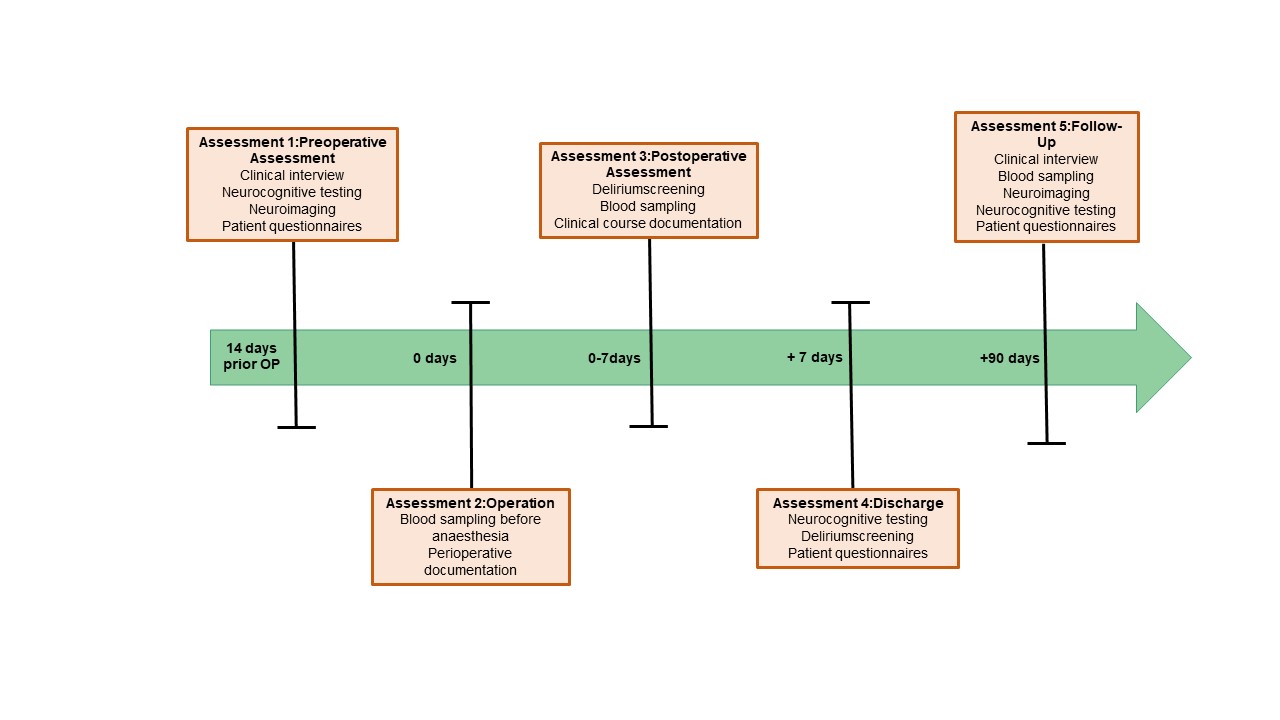

Supplement: Supplementary file 3 — Supplemental Figure 1 [file 41398_2021_1304_MOESM3_ESM.jpg]

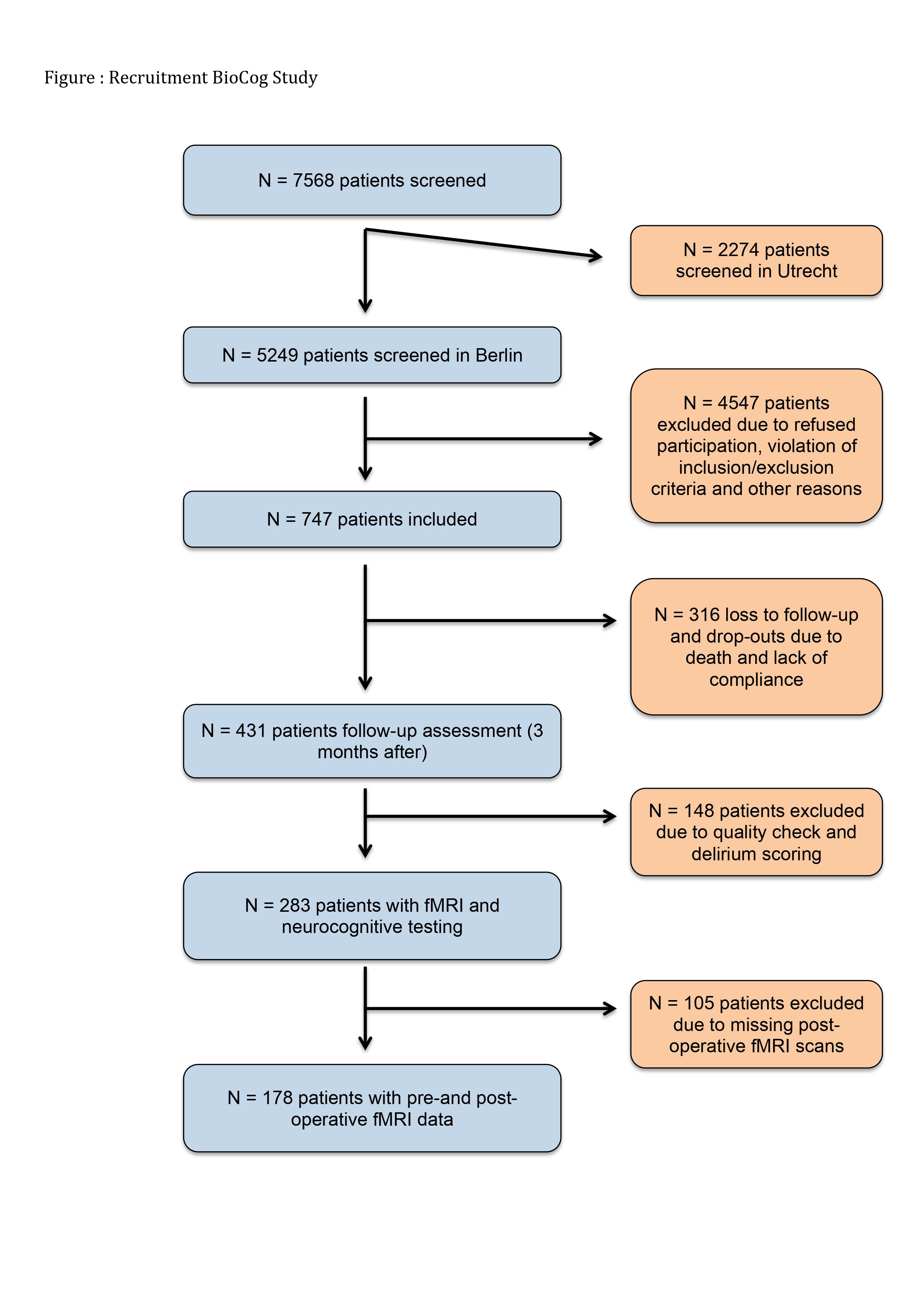

Supplement: Supplementary file 4 — Supplemental Figure 2 [file 41398_2021_1304_MOESM4_ESM.jpg]
